# Influences on help-seeking for serious mental illness in Dhaka, bangladesh: a mixed-methods study

**DOI:** 10.1007/s00127-025-03012-0

**Published:** 2025-10-31

**Authors:** Sagar Jilka, Bulbul Siddiqi, Cathy Winsper, Georgios Bouliotis, Ursula M. Read, Tanjir Soron, Azmery Shammin, Simon J. Smith, Dafne Morroni, Helal Uddin Ahmed, Olayinka Omigbodun, Swaran Preet Singh

**Affiliations:** 1 Warwick Medical School, University of Warwick, Coventry, CV4 7AL UK; 2 Institute of Psychiatry, Psychology and Neuroscience, King’s College London, London, UK; 3 Warwick Centre for Global Health, University of Warwick, Coventry, UK; 4 North South University, Dhaka, Bangladesh; 5 School of Health and Social Care, University of Essex, Colchester, UK; 6 Telepsychiatry Research and Innovation Network Ltd, Dhaka, Bangladesh; 7 The National Institute of Mental Health (NIMH), Dhaka, Bangladesh; 8 Department of Psychiatry, College of Medicine, University of Ibadan, Ibadan, Nigeria; 9 Coventry and Warwickshire NHS Partnership Trust, Coventry, UK

**Keywords:** Help-seeking, Serious mental illness, Low-and-middle-income-countries, Care pathways, Traditional and faith healers, Improving access

## Abstract

**Purpose:**

Early intervention can improve mental health outcomes for people living with serious mental illness (SMI). Understanding what factors influence patients’ health help-seeking decisions are important in low and middle-income countries (LMICs) where resources and outcomes are poor, particularly in slums, to help inform targeted intervention approaches.

**Methods:**

A concurrent triangulation mixed methods study conducted in Dhaka, Bangladesh, using a quantitative pathway to care questionnaire with individuals from a local slum (Korail) attending the National Institute for Mental Health (NIMH), a specialised hospital for mental health services. Qualitative interviews were conducted with people with SMI and family caregivers living in Korail.

**Results:**

28,896 patients attended NIMH between 24th September 2022 and 25th September 2023 and only 0.11% (*n* = 33) came from the Korail slum. 46% had previously seen a faith or traditional healer. Qualitative interviews with people with SMI and caregivers in Korail showed that spiritual possession was among several perceived causes of SMI. Participants also percieved pharmacological treatment as expensive and potentially harmful. However participants also reported a lack of knowledge about specialist mental health facilities and spending considerable funds and resources on healers and private medical providers in the hope of cure.

**Conclusions:**

Help-seeking among families living in slums in Dhaka is pluralistic, with complex influences on treatment choice. Understanding help-seeking behaviour and care pathways is crucial to design an equitable health system and improve access to effective mental health care.

**Supplementary Information:**

The online version contains supplementary material available at 10.1007/s00127-025-03012-0.

## Introduction

Help-seeking for serious mental illness (SMI) in low- and middle-income countries (LMICs) is pluralistic. Individuals with SMI, with their family, seek treatment from a diverse range of sources, including biomedical facilities and traditional and faith healers (TFHs) [1]. TFHs are often the initial, and sometimes the only, port of call [2]. For instance, the preferred first contact for mental disorders is TFHs for 30% of adults in Bangladesh, although similar proportions of patients with SMI also visit private psychiatry services [3]. Many factors influence the pathways families take to seek mental health (MH) care, including cost, availability, and quality of care, as well as beliefs about mental illness [4]. This can result in delays and difficulties in accessing biomedical treatment [5]. Delays in seeking biomedical care can result in prolonged duration of untreated mental illness (DUI) which can lead to poorer prognosis and increased treatment costs [6, 7].

Understanding these pathways can identify barriers to effective treatment and inform interventions to improve access and improve quality of care. WHO advocates for interventions to close the ‘treatment gap’ (i.e., the difference between those in need of treatment and those receiving it [8]). Approaches to close the gap include integration of MH into primary care and multi-sectoral approaches building on community assets, including collaboration with TFHs [9–11]. Whilst there have been longstanding calls for such collaboration, there are known barriers, including distrust between biomedical practitioners and TFHs and resource limitations in the health sector [4]. Most research has been conducted in African countries with fewer studies in Asia [12]. There have also been fewer studies of use of TFHs in urban areas, where MH services tend to be more accessible.

This study seeks to understand the pathways to MH care for people with SMI living in the Korail *basti* (*basti* meaning slum) in Dhaka using quantitative (survey) and qualitative (interview) data. Despite previous work showing pluralistic help seeking in Dhaka [3], pathways to MH care for people with SMI living in slums is not known. This study is part of a larger National Institute of Health Research (NIHR) funded project (TRANSFORM) [1] which aims to improve outcomes of persons with SMI through better access to effective biomedical care by developing an innovative collaborative care model between TFHs, MH professionals, and community health workers (CHWs) in Bangladesh and Nigeria. This paper reports on data from Bangladesh only. To support the overall objective of the TRANSFORM study, we aimed to: (1) quantitatively idenitfy the care pathways of people seeking biomedical care at the National Institute of Mental Health (NIMH), Dhaka, Bangladesh; (2) determine if DUI was associated with sociodemographic factors in those attending biomedical care, and (3) qualitatively explore the influences on care pathways from the perspective of individuals with SMI and their caregivers.

## Methods

### Design

This was a concurrent triangulation mixed methods study involving a longitudinal 12-month survey (24/09/2022 to 25/09/2023) of patients seeking help at NIMH, a tertiary level hospital and specialist MH care provider in Dhaka, Bangladesh. This was accompanied by in-depth interviews with people with SMI and their families, from the Korail slum.

### Study sites

Study participants, for both the encounter quantitative questionnaire (described below) and qualitative phases lived in Korail slum in Dhaka, Bangladesh. Korail is one of the largest slums in Bangladesh, between 180 and 220 acres [13]. Most of Korail’s population (200,000–400,00) [14, 15] live in poverty, work in low-income and insecure jobs with limited access to healthcare services and surrounding major roads [16].

Participants were recruited at NIMH, Bangladesh’s leading public MH institution dedicated to MH policy formation, research and training of MH professionals. The facility is located 7.3 km from Korail with 400 in-patient beds. A comprehensive overview of Bangladesh’s health system can be found in [17, 18] and in the Supplementary Material.

### Data collection

#### Quantitative

 A semi-structured interviewer-administered questionnaire was developed based on the Gater pathways encounter form to gather systematic information about the sources of care used by participants before they came to NIMH [19, 20]. The structured questionnaire was developed in line with previous studies and the WHO pathway study methodology and its pathway encounter form [19, 21–23].

All patients attending for consultation at NIMH were screened using three questions prior to being invited to complete the encounter questionnaire:

1. Have you come from the Korail Basti?
2. Have you been referred by a traditional and faith healer?
3. Have you ever seen a traditional and faith healer for mental health treatment?

Participants who answered yes to questions one or two were consented and invited to complete the encounter form. The encounter form asked questions about the participant’s diagnosis, time of illness onset (in months), first symptom(s) experienced by the patient, place(s) of care visited, source(s) and dates of referral to NIMH, treatment received, symptom(s) that prompted the decision to seek help, who took the decision to seek further care, and whether they are maintaining regular follow up. It was modified and translated into Bengali, the primary language in Bangladesh, by the Dhaka-based research team.

#### Qualitative

We conducted modified focused ethnography in Korail using semi-structured interviews with people with SMI and family caregivers. Before data collection commenced, community entry and engagement activities were conducted. This included regular site visits by members of the study team, meetings with key stakeholders and the formation of a local steering committee. People living in Korail were recruited as community liaison workers (CLWs) to act as guides and intermediaries to disseminate information about the study within the communities and support identification and recruitment of research participants. Interviews were conducted in Korail either in family homes or at a designated field office rented for the project. Interviews were conducted in Bengali and audio recorded with the consent of the respondents. Our topic guide was informed by a combined framework that considered (a) explanatory models of mental illness (local attributions and help-seeking logics), (b) dimensions of access (e.g., perceived acceptability, affordability, availability), and (c) filters along pathways to care (e.g., family/lay networks, TFHs, primary and specialist services). Constructs operationalised in questions included perceived causes, stigma and anticipated discrimination, trust/mistrust in providers, costs and perceived harms/benefits of treatments, decision-making within households, and practical navigation barriers, as per the activities of work package 1 outlined in our protocol paper [1]. Interviews with persons with SMI and caregivers were conducted separately wherever feasible; brief joint debriefs were held only on participant request. Parallel, role-specific topic guides mapped to the same constructs.

### Sampling and recruitment

#### Quantitative

We screened all patients visting the outpatient department at NIMH between 24th September 2022 to 25th September 2023. Patients were often accompanied by their caregivers/family members. Encounter forms were interviewer-administered to patients at NIMH out-patient clinics immediately after screening. The patient was the primary respondent. When an accompanying family caregiver was present, interviewers invited caregiver input to corroborate dates and service names; the patient’s account was prioritised as the reference. If the patient was unsure and no caregiver was available, items were coded as ‘missing’ rather than inferred. Fieldworkers received protocolised training in neutral probing, documenting uncertainty, and recording discordant responses. Patients who answered ‘yes’ to either coming from Korail or being referred from a TFH from Korail were invited to complete the quantitative encounter survey.

#### Qualitative

Research teams used a combination of purposive and snowball sampling. Potential participants, caregivers and people with SMI were identified through community gatekeepers and support from CLWs. Together, the research team and CLWs visited mosques, clinics, drug sellers and family homes and met with potential participants in Korail. The research team and CLWs were trained to identify people with possible symptoms of SMI. Potential participants were then assessed by the psychiatrist from the TRANSFORM team in Bangladesh (TS). Qualititave data collection took place between June - November 2022. Snowballing proceeded from initial ‘seeds’ identified with gatekeepers and CLWs: participants subsequently referred other households affected by SMI. We recruited both persons with SMI and caregivers; dyads were included when feasible but were not required. CLWs were CLWs with prior experience of mental health, but were trained on recognition of probable SMI, safeguarding and confidentiality, approaching households respectfully, and referral procedures. To minimise inadvertent stigmatisation, recruitment materials used neutral language (‘health and care-seeking study’), interviews were conducted privately (home room with door closed or field office), and CLWs were trained to avoid labels and to protect confidentiality within close-knit areas.

### Data analysis

#### Quantitative

 Descriptive analyses were performed to characterise the socio-demographic and clinical characteristics of patients completing the encounter form. Patients (with support from their caregivers), were asked to describe where the patient was first taken for treatment. This information was categorised into ‘traditional’ or ‘biomedical’ sources by the Dhaka research team after reviewing the descriptions of where participants were taken for their first treatment (see supplementary material for our categorisation). The question regarding ‘who initiated the first contact’ was categorized into the following categories adapted from a previous study [24], as ‘healer’, ‘family/relatives’, ‘friends’, or ‘self-referral’. DUI was defined as the time in months between the onset of initial changes in behaviour or symptoms noticed to when a first contact for help was made. Patients and caregivers were interviewed by the research team and asked, ‘when they first felt that they needed help (specify age)’, ‘duration of initial change in behaviours or symptoms noticed’ and ‘how long ago did the first contact happen’. DUI was then calculated after combining information from the interview. We converted this as a dichotomous variable of short (≤ 6 months) versus long (> 6 months) duration [25]. We used chi^2^ tests with Yates’ correction, as our variables were not all dichotomous, to determine whether there are statistically significant differences in duration (DV) across the following independent variables; diagnosis, first place of care, or initiator of care, and demographic variables (age, as a categorical variable and gender). Statistical significance was evaluated at a *p* value of < 0.05 using two-sided tests.

#### Qualitative

Interviews recordings were transcribed verbatim in Bengali, before the transcripts were translated into English. Translated transcripts were checked and corrected by researchers fluent in English and Bengali. Data were analysed by social science researchers in Bangladesh and the UK (BS, UR) following a reflexive critical thematic approach to reflect on dominant assumptions within a culture and actively engage with complexity and uncertainty [26]. This approach recognises the positionality of the researcher and the influence on the ways in which knowledge is constituted [26]. Data analysis was iterative. The first stage involved data familiarisation through repeated reading of the transcripts to manually identify initial codes. A codebook was developed from the initial codes which was refined through discussion among the qualitative research team members and further close reading of the transcripts. All transcripts were then coded by three researchers using NVivo 20. Codes were then grouped into deductive themes informed by the research questions and inductive themes identified within the findings. Themes were further refined through discussion with research team members with qualitative research expertise in Bangladesh, UK and Nigeria. Throughout the analysis process, the research team engaged in reflection on the positionality, experience, professional training and values of the team members (UR is a White British female, BS is a Bangladeshi male) and the ways in which this influenced the interpretation of the data. This enabled analysis of experiences, values and beliefs expressed in the interviews from positions as insider and outsider, grounded in a critical realist orientation to the data as situated within a particular political, historical and sociocultural context [27]. We monitored emergent codes across interviews and judged *thematic sufficiency* when no new codes of relevance to our constructs emerged in the final interviews for each role. In keeping with reflexive thematic analysis, our aim was conceptual depth and coherence rather than numerical ‘data saturation’.

## Results

### Quantitative results

#### What proportion of participants from the slum site seek medical help?

Between 24th September 2022 and 25th September 2023, we screened 100% of the total 28,896 patients who attended NIMH. Of them, 46% (*N* = 13,269) had at some point, seen a TFH for help in the past. We found that less than 1% (*n* = 33) came from Korail.

Of the thirty-three patients from Korail who completed the encounter form, the mean age of participants was 32.5 (± 12.3) with a slightly greater proportion of men (55%). All patients had an SMI - the majority had a diagnosis of schizophrenia (45, *N* = 15), followed by bipolar disorder (30%, *N* = 10), Major Depressive Disorder (MDD) (12%, *N* = 4), conversion disorders (6%,*N* = 2) and substance use disorders (6%, *N* = 2). Participant’s median duration of illness was 42 months, with 54% (*N* = 18) seeing a TFH as their first source of care, 42% (*N* = 14) seeing a biomedical practitioner as their first source of care, and one participant reporting seeing both. This first point of care was largely initiated by family/relatives (e.g., parents, siblings, spouse) (76%), followed by friends (9%), healers (9%), or self-initiated (6% (*N* = 2)).

We found a statistically significant difference between the 6 month DUI threshold and the person who initiated the first care (χ² = 13.32, *df* = 3, *p* = 0.004) but found no statistically significant differences when comparing DUI with age, gender, place of care and the diagnosis categories.

#### What proportion of participants had been referred from, or sought help from, a TFH or CHW?

42% of the 33 participants coming to NIMH came directly (without referral) from Korail, whilst 58% (*N* = 19) were referred. Of these 19 patients, eight (42%) had been referred from either a TFH, and 8 (42%) had been referred from a biomedical source.

### Qualitative results

Twenty-three participants took part in semi-structured interviews (people with SMI: *N* = 7 (mean age = 35 ± 16.12); caregivers: *N* = 16 (mean age = 48.56 ± 13.3). Supplementary Material Table III provides demographics information of the samples. Sampling sought *information power* within a narrowly defined phenomenon (help-seeking for SMI in Korail), prioritising depth over breadth. None of the participants were included in the quantitative NIMH survey. Factors influencing choice of treatment included: (i) experimentation with various forms of treatment, both biomedical and TFH in the hope of a cure; ii) distrust of medical practitioners and experiences of harmful treatment and (iii) a lack of knowledge of specialist MH services.

### Theme 1a: experimentation and the search for a cure

Qualitative findings revealed that although the influence of *jinn* or other spiritual factors, such as evil eye, was often suspected as a cause of mental illness, in practice familes commonly experimented with different treatment options including religious teachers (*hujur)*, *kabiraj*, plant-based medicines, biomedical doctors, and private drug sellers. The choice of treatment was usually based on the advice of family, friends, neighbours and, sometimes, religious leaders.

Although four out of seven participants with SMI had been first taken to a TFH, others sought help from a *kabiraj* or *hujur* when symptoms did not improve with medical treatment. A caregiver explained how he treated his mother in various biomedical clinics, hospitals and private doctors. After five months of medication, his mother’s condition did not improve. He tried a private MH hospital, but again, there was little change. Finally, he decided to try a *kabiraj* who advised that his mother was afflicted by a spirit:

‘He [*kabiraj*] said she [mother] was possessed by a bad spirit (*alga dosh*) I then said to the *kabiraj*, “Okay. Whatever you want to give, hand it over, I will try by the grace of Allah”. He gave *pani pora* and holy oil [water and oil that have had the Qur’an recited over them]. He said, “Massage the oil and drink the water. Everything will be fine In Sha Allah” (Male caregiver).

This illustrates the complex, non-linear help-seeking pathways followed by families seeking treatment for family members with MH problems. Families usually try various methods simultaneously or sequentially in the search for an effective cure, as described by this woman caring for her aunt:

‘Now I’ve gone to *kabiraj*. He recites *ayahs* [verses from the Qur’an] with the name of Allah. Now we’re doing that too. Then we’re giving her medicines after taking her to the hospital.’ (Female caregiver).

Participants were generally disappointed by the failure to obtain a cure, despite trying multiple treatment options. One participant with SMI shared that his grandmother, who is also a traditional healer, suggested that he visit various healers and doctors but this was ultimately unsuccessful:

*“She [his grandmother] suggested to take me to a traditional healer. She took me to traditional healer and when we found out that it did not work*,* they took me to a doctor”* (25 year old male patient).

The lack of symptom improvement creates frustration and feelings of hopelessness resulting in some families discontinuing treatment. Limited effectiveness and the greater treatment cost seem to act as the determining factors for withdrawing and selecting other treatment methods. However, participants often resume help-seeking if they hear of an alternative option with the hope of finding a cure for their loved ones.

### Theme 1b: explanatory models and treatment logics

Participants articulated co-existing models of illness, spirit-related (e.g., jinn, evil eye) and biomedical, which shaped the perceived appropriateness and sequencing of help (e.g., hujur/kabiraj vs. private doctor or hospital). Families often toggled between models, especially when perceived ‘cure’ was not achieved. Across themes, anticipated or enacted stigma, precarious income and costs of repeated consultations, and worries about discrimination at work or school constrained choices and continuity of care.

### Theme 2: distrust of biomedical practitioners and experiences of harmful treatment

Caregivers described spending significant amounts of money on private medical treatment, sometimes without any successful outcome, or even harmful effects. A caregiver stated her frustration with the cost which had led her to stop seeking treatment:

“Now I don’t want to get any more treatment. I don’t even have that much money. A lot of money is needed to treat her” (Female caregiver).

Unsurprisingly, there was evidence of some mistrust towards medical doctors. This caregiver, for example, expressed concern that some doctors were only motivated by greed:

‘All they care about is money, don’t you understand? (Female caregiver).

Such mistrust can motivate people to seek help from TFHs or spiritual teachers instead.

Several participants also described harmful or unpleasant experiences of medical treatment leading to abandoning one source of treatment for another:

*“Those medicines made my son mad; everyone advised him to stop those medicines*,* which had a bad reaction*,* and his eyes protruded. Too much medicine was not suiting my son’s brain. Then*,* people in this area asked me not to take these medicines. Then what should we do? One person gave the news of a good doctor who had a chamber at Mymensing. We heard that patients were getting well from him. From there on*,* we have been taking medicine from there for two to three years”* (Female caregiver).

Constraints on time and finances, coupled with their unpredictable income sources, often prevented regular or extended visits to medical facilities. Experiences of harmful medical treatment and high treatment costs could lead some to discontinue treatment altogether and undermine their faith in the effectiveness of biomedical treatment for mental illness.

### Theme 3: limited awareness of specialist mental health services

Although participants were aware of the possibility of medical treatment for mental illness and had made use of various general public and private medical practitioners, surprisingly, participants had limited knowledge about specialist MH services at NIMH. We found that only a few participants went to NIMH to seek help. One reason is the lack of awareness of MH service providers. However, only one participant mentioned that he went to a MH hospital due to experiencing MH problem. As he stated:

*‘‘I went to a mental health hospital [NIMH] as I have a mental health problem’’ (Male patient)*.

Many also believed that treatment at NIMH was expensive despite being comparatively more affordable:

‘‘*When we went to a hospital in Agargaon [NIMH] with ten taka ticket and transportation cost. Whatever medicines they gave me there*,* others have to buy. At first*,* it was very hard with money’’* (Female Caregiver).

Although seeing a doctor at NIMH is inexpensive (only 10 BDT equivalent to 0.083 USD), transport and medication costs make it difficult for caregivers. Many caregivers also spend a lot of money before going to biomedical services, as a caregiver reported, “I didn’t understand at the beginning. I wasted a lot of money going to kabiraj”. This also shows their preference towards TFHs over biomedical services due to the lack of awareness of biomedical services.

## Discussion

Help-seeking for MH in Bangladesh is pluralistic, so understanding use of psychiatric services and understanding why and how families chose services may help to produce meaningful and sustainable change in access to care and potentially improve the treatment of SMIs in LMICs. Our study indicated that only 11 out of every ten thousand patients visiting NIMH came from the Korail slum, and many (46%) had previously sought help from TFHs. Of the few people who came to NIMH (*n* = 33), 58% (*n* = 19) were referred from either TFHs (42%, *n* = 8) or had been referred from a biomedical source (42%, *n* = 8). Qualitative interviews provided further detail on help-seeking by poor families living in Korail including experimental and pluralistic approaches to help-seeking among both biomedical practitioners and TFH, distrust of biomedical practitioners and experiences of harmful biomedical treatment. Qualitative findings revealed that participants had limited awareness of NIMH as offering specialist treatment for mental illness and misconceptions about the cost.

Our findings align with observations from other LMICs. Notably, 46% of our study participants sought help from TFHs before approaching biomedical services. This prevalence is considerably higher than the 22% reported in earlier studies conducted in Dhaka [22, 23] although the sample size in these studies is considerably smaller (*N* = 50). Our findings are, however, reflective of research in LMICs where TFHs play a pivotal role in the treatment of mental illness [2, 3]. For instance, previous studies reported high use of TFHs, with figures ranging from 23% (Ghana; [5]) to 69% (India; [28]).

The comparison of these figures highlights the significant influence of socio-cultural factors on health-seeking behaviours, suggesting that interventions in LMICs must be tailored to address these unique cultural and systemic contexts to enhance the efficacy of MH care delivery. Importantly, we also found despite a large proportion of participants visiting TFHs, many (42%, *N* = 14) reported seeing a biomedical practitioner exclusively as their first source of care. This is in line with previous work in Korail [3] who found 30% of patients visited a biomedical practitioner as their first source of care.

### Implications of pluralistic help-seeking

The pluralistic help-seeking patterns identified in our study, where 46% of participants initially turned to TFHs, have important implications for MH outcomes in Dhaka. Such diversity in help-seeking behaviour, directly impacts the duration of untreated illness (DUI), which has been associated with poorer prognostic outcomes (i.e., severe symptoms, decreased treatment responsiveness [6, 7]). Pluralistic pathways often result in delayed engagement with biomedical services, which extend the DUI, thus exacerbating the severity of mental disorders over time [2].

As shown in other studies in Bangladesh and other Muslim majority societies, belief in jinn and other spiritual factors such as evil eye as causative factors may lead families to seek spiritual treatments for mental illness [5, 22].

This is compounded by a distrust of medical practitioners and treatments and the perceived high costs of biomedical treatment, despite substantial expenditures on traditional healing practices, a paradox also observed in another study at NIMH [3].

Understanding the factors that impact on help-seeking is crucial for developing MH interventions that are not only culturally and contextually appropriate but also effective in reducing DUI and improving overall MH outcomes. Collaborative efforts between biomedical health services and TFHs, as suggested by the TRANSFORM project [1] and other studies in LMICs [29], could serve as a viable approach to engage communities, enhance trust, and facilitate access to MH care.

### Equitable care and health system improvements for disadvantaged groups

Our study underscores the critical importance of equitable and quality care that is culturally sensitive in the design and implementation of MH interventions. To be effective, interventions must respect and incorporate local beliefs and practices, acknowledging the role these play in the community help-seeking behaviour. Training programs for healthcare providers should enhance their ability to engage respectfully and effectively with patients whose beliefs about illness and healing may differ from conventional medical views. In addition, the quality of MH care should be improved, to reduce coercive practices and address patients’ concerns about the effects of pharmaceutical treatments, which are often experienced as disabling and unpleasant [2, 29]. This can lead to discontinuation of treatment, particularly in the long term.

At a system level, improvements are needed to facilitate easier access to MH services for people living in deprived communities such as urban slums. This includes developing and implementing referral systems that bridge TFH with public and private biomedical services. Such systems could help streamline the care pathway, ensuring that patients receive timely, affordable, accessible, effective and compassionate care. Additionally, policy reforms should aim to enhance the infrastructure and resources available at MH facilities, making them more accessible to underserved populations. By bringing together a multi-sectoral approach across different sources of care within and beyond the health system [8], a more inclusive and effective framework for MH care can be created that addresses the diverse community needs.

### Limitations and future directions

The study has several limitations. First, the data collection was confined to patients attending a single institution - the National Institute of Mental Health -which may limit the generalisability of findings to other regions (e.g., rural areas) or health care settings in Bangladesh. We caution against generalising Korail-specific estimates beyond urban Dhaka tertiary attenders; patterns may differ in other slums and rural areas. Our findings showed that there are other institutions (i.e., general hospitals) offering MH care, so patients accessing care at this facility may not be representative of the broader population. Additionally, the specific focus on individuals from Korail might not capture the diversity of experiences and behaviours prevalent across other regions in Bangladesh. The insights gained may thus, reflect the unique socioeconomic and cultural contours of this specific community, which could differ significantly from other communities. Furthermore, quantitatively, we only asked if patients had previously seen a TFH prior to attending NIMH, and not whether they had seen a biomedical practitioner. This may suggest that many patients had *only* seen a TFHs prior to NIMH, whereas the qualitative data show that in fact families in Bangladesh commonly use a mixture of biomedical practitioners and healers. However, our focus was on understanding the proportions of patients who previously visited a TFH to inform the development of our intervention. Moreover, the reliance on self-reported data for understanding the care pathways, may introduce biases related to memory or social desirability. Participants may under or over report their reliance on TFHs or their engagement with biomedical services due to perceived stigma or expectations from the research team. Furthermore, we were not able to conduct qualitative interviews with a sub-sample of Korail based participants during this study; we are now following up these patients to deepen pathway triangulation in a subsequent report. Finally, this study was purposefully centred service-user and caregiver perspectives; complementary TRANSFORM work engages TFHs and clinicians. We note the absence of provider interviews here as a limitation and opportunity for triangulation. Our mixed methods data were integrated as while the survey quantified pluralistic pathways (46% TFH contact before NIMH; family/relatives as initiators associated with longer DUI), while qualitative data illuminated *why* these patterns arise; household decision-making anchored in lay networks, experimentation after perceived harms or non-response, and affordability/trust calculations.

Future research could employ longitudinal designs to track changes in help-seeking behaviours over time, regarding evolving socio-economic conditions and healthcare interventions. This would offer insights into the dynamics of help-seeking patterns and the long-term efficacy of interventions aimed at improving access to effective care. There is also a pressing need to explore interventions that facilitate collaboration between traditional healing practices and biomedical care. Future studies should examine whether collaboration can effectively reduce DUI, improve treatment adherence, and ultimately enhance MH outcomes.

## Conclusions

Help seeking in Bangladesh is pluralistic, but health systems are not organised to provide appropriate care services to patients with MH problems. Understanding of MH help-seeking behaviour and care pathways are crucial to design an equitable health system and plan interventions to improve access to care. Most people with SMI are likely to need biomedical treatment so interventions are required to be both accessible and acceptable. This should be accompanied by interventions addressing social and psychological needs, particularly for marginalised and disadvantaged communities (i.e., people living in slums). Interventions that encourage collaboration between healers and biomedical services must ensure a respectful approach to build mutual trust.

## Supplementary Information

Below is the link to the electronic supplementary material.

Supplementary Material 1

## Data Availability

Data is available upon request from the corresponding author.
