# Supplementary material for: Influences on help-seeking for serious mental illness in Dhaka, bangladesh: a mixed-methods study
Source: Soc Psychiatry Psychiatr Epidemiol. 2025 Oct 31;61(2):357–65. doi: 10.1007/s00127-025-03012-0 (PMC12948840; doi:10.1007/s00127-025-03012-0)
Supplement: Supplementary file 1 — Supplementary Material 1 [file 127_2025_3012_MOESM1_ESM.docx]

**Title**: Influences on help-seeking for serious mental illness in Dhaka, Bangladesh: a mixed-methods study

**Authors**: Sagar Jilka^1,2,3^, Bulbul Siddiqi^4,^, Cathy Winsper^1,3^, Georgios Bouliotis^1^, Ursula M Read^5^, Tanjir Soron^6^, Azmery Shammin^6^, Simon J Smith^1,3^, Dafne Morroni^1,3^, Helal Uddin Ahmed^7^, Olayinka Omigbodun^8^, Swaran Preet Singh^1,3,9^

**Author Affiliations:**

^1^ Warwick Medical School, University of Warwick, Coventry, UK

^2^ Institute of Psychiatry, Psychology and Neuroscience, King’s College London, UK

^3^ Warwick Centre for Global Health, University of Warwick, Coventry, UK

^4^ North South University, Dhaka, Bangladesh

^5^ School of Health and Social Care, University of Essex, UK.

^6^ Telepsychiatry Research and Innovation Network Ltd, Dhaka, Bangladesh

^7^ The National Institute of Mental Health (NIMH), Dhaka, Bangladesh.

^8^ Department of Psychiatry, College of Medicine, University of Ibadan, Ibadan, Nigeria

^9^ Coventry and Warwickshire NHS Partnership Trust, Coventry, UK

**Corresponding author:**

Professor Swaran P. Singh

Email: [s.p.singh@warwick.ac.uk](mailto:s.p.singh@warwick.ac.uk)

Full postal address: Warwick Medical School, University of Warwick, Coventry, CV4 7AL, UK

**Supplementary Material**

***Study sites***

Participants completing the quantitative survey were recruited at NIMH. NIMH is Bangladesh's leading public institution dedicated to the treatment of mental health problems, along with mental health policy formation, research and training of mental health professionals. It has seven departments of psychiatry sub-specialities. The facility is located 7.3 km from Korail. It serves as one of the cheapest options for mental health treatment and employs over 300 personnel, including psychiatrists, nurses and psychologists, to deliver out-patient, in-patient and emergency medical services for child and adult mental disorders. NIMH has a total of 400 in-patient beds; 70% of these are non-fee-paying government-funded beds and the rest are government determined fee-paying beds. In-patient and out-patient admissions range across all units, including adult psychiatry and community and social psychiatry. In 2019, 58 846 patients were seen in the out-patient department. Access to out-patient services are very low cost (only 10 taka (£0.07/$0.08) for registration) and medication for patients is provided free of charge for two weeks. The cost for in-patient treatment is also very low, and medication is provided free of cost while an inpatient.

Alongside NIMH and government services, there are numerous private drug sellers in Korail who are commonly perceived as medical professionals and used as the first line of health advice and treatment. When common ailments persist or worsen, people may seek medical care from private doctors who are usually located outside the slum, or from nearby hospitals. A comprehensive overview of the Bangladesh health system can be found in (Hasan et al., 2021a, 2021b).

*Information related to standardising the first place of care:* The following descriptions were categorised as ‘Traditional’ sources of care: “Telpora, panipora, manot (oath to pay money in the name of god), kabiraj/kobiraj, traditional healing (Jhar Phukh, Pani pora, (oil, water treatment)”. The following descriptions were categorised as ‘Biomedical’ sources of care: “Doctor, Mental Treatment, Prescribed medicines, Medicine, injection”.

| Supplementary Table I. Contingency table of length of DUI and who initiated first contact. | | | | |
| --- | --- | --- | --- | --- |
|  | Family/relatives | Friends | Healer | Self |
| Long DUI | 24 | 1 | 3 | 2 |
| Short DUI | 1 | 2 | 0 | 0 |

| Supplementary Table II. Profile of the thirty-three participants from our NIMH survey | | | | | | | |
| --- | --- | --- | --- | --- | --- | --- | --- |
| **Ppt** | **Age** | **Gender** | **Diagnosis** | **DUI (months)** | **Description of First Symptoms** | **First Place of Care** | **First Care Initiated By** |
| 1 | 26 | F | Schizophrenia | 13 | Hallucination | Traditional | Healer |
| 2 | 25 | F | Schizophrenia | 144 | Biting people, Running to and fro | Traditional | Healer |
| 3 | 67 | F | Schizophrenia | 270 | Dizziness | Traditional | Family/relatives |
| 4 | 20 | F | BMD | 42 | Anxiety, Could not recognise Family members | Traditional | Family/relatives |
| 5 | 42 | F | MDD with ASD | 336 | Sleep disorder | Traditional | Family/relatives |
| 6 | 23 | M | Susbtance Use Disorders | 0.5 | Irrelevant talking, Disorganized speech | Traditional | Family/relatives |
| 7 | 20 | M | BMD | 1 | Short Sleeping | Traditional | Friends |
| 8 | 30 | F | Schizophrenia | 180 | Abnormal Attitude | Traditional | Friends |
| 9 | 48 | F | Schizophrenia | 12 | Mental Sick | Traditional and Biomedical | Family/relatives |
| 10 | 21 | M | MDD | 16 | Vomiting, Migraine | Biomedical | Family/relatives |
| 11 | 50 | F | BMD | 8 | Abnormal Activities | Traditional | Self |
| 12 | 30 | M | Substance Use Disorders | 36 | Abnormal Activities | Biomedical | Family/relatives |
| 13 | 25 | M | Schizophrenia | 60 | Smiling | Traditional | Healer |
| 14 | 38 | F | BMD | 24 | Tension, Extra talking, Objurgate | Biomedical | Family/relatives |
| 15 | 18 | M | BMD | 24 | Abnormal Activities | Traditional | Family/relatives |
| 16 | 41 | M | Schizophrenia | 50 | Staying detached from everyone and remains silent | Biomedical | Family/relatives |
| 17 | 47 | F | BMD (M)/1st Episode | 2 | Over talking, Sleeplessness | Biomedical | Friends |
| 18 | 34 | M | BMD | 186 | Unusual silence | Biomedical | Family/relatives |
| 19 | 22 | M | Schizophrenia | 120 | Unusual movement and behaviour | Biomedical | Family/relatives |
| 20 | 26 | M | Schizophrenia | 156 | Brain affect after typhoid | Traditional | Family/relatives |
| 21 | 25 | M | Schizophrenia | 72 | Speechlessness, Sleeplessness, Abnormal Activities | Biomedical | Family/relatives |
| 22 | 30 | M | Schizophrenia | 24 | Sleeplessness, Aggressive | Traditional | Family/relatives |
| 23 | 19 | M | Schizophrenia | 18 | Disorganized talking, Disorganized behaviour | Biomedical | Family/relatives |
| 24 | 60 | F | Schizophrenia | 65 | Disorganized behaviour, Disorganized talking | Biomedical | Family/relatives |
| 25 | 30 | M | Conversion disorder | 55 | Disorganized behaviour, Memory loss | Traditional | Family/relatives |
| 26 | 21 | M | MDD with GAD | 30 | Short memory loss, Headche, Decrease appetite, Insomnia | Biomedical | Self |
| 27 | 26 | F | Schizophrenia | 50 | Disorganized behaviour, Disorganized talking, Insomia, Aggressive | Traditional | Family/relatives |
| 28 | 50 | F | Bipolar disorder | 444 | Aggressive, Insomnia, Disorganized behaviour, Disorganuzed talking, Overactivity | Traditional | Family/relatives |
| 29 | 35 | M | Conversion Disorders | 240 | Senseless, Aggressive, Memory loss | Biomedical | Family/relatives |
| 30 | 27 | F | OCD | 60 | Repeated work, Aggressive | Biomedical | Family/relatives |
| 31 | 42 | M | Schizophrenia | 24 | Aggressive, Insomnia, Negative behaviour | Biomedical | Family/relatives |
| 32 | 30 | M | BMD | 24 | Aggressive, Insomnia | Traditional | Family/relatives |
| 33 | 26 | F | BMD | 37 | Aggressive, Insomnia | Traditional | Family/relatives |

| Supplementary Table III. Profile of the participants from our qualitative component | | | |
| --- | --- | --- | --- |
| Demographic characteristics |  | People with SMI  (n=7) | Caregivers (N = 16) |
| Gender | Female | 4 | 14 |
|  | Male | 3 | 2 |
| Age | 18-30 | 3 | 2 |
|  | 31-45 | 2 | 6 |
|  | 46-60 | 1 | 6 |
|  | 61-80 | 1 | 2 |
|  | 81+ | - | - |
| Ethnicity | Bengali | 7 | 16 |
| Religion | Christian | 0 | 0 |
|  | Muslim | 7 | 16 |
|  | Traditional religion | 0 | 0 |
| Education | Primary | 3 | 4 |
|  | Secondary | 2 | 1 |
|  | Tertiary | 1 | 0 |
|  | Uneducated | 1 | 11 |
| Marital status | Married | 3 | 12 |
|  | Single | 2 | 0 |
|  | Divorced | 2 | 0 |
|  | Widowed | 0 | 4 |
| Occupation | Self-employed | 4 | 7 |
|  | Housewife | 1 | 3 |
|  | Unemployed | 2 | 6 |


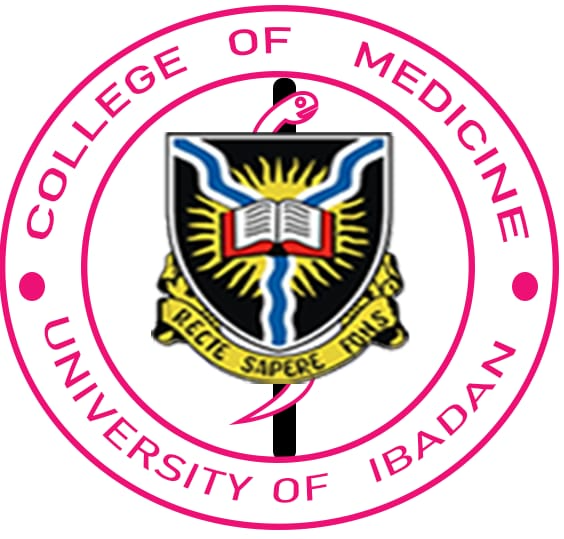

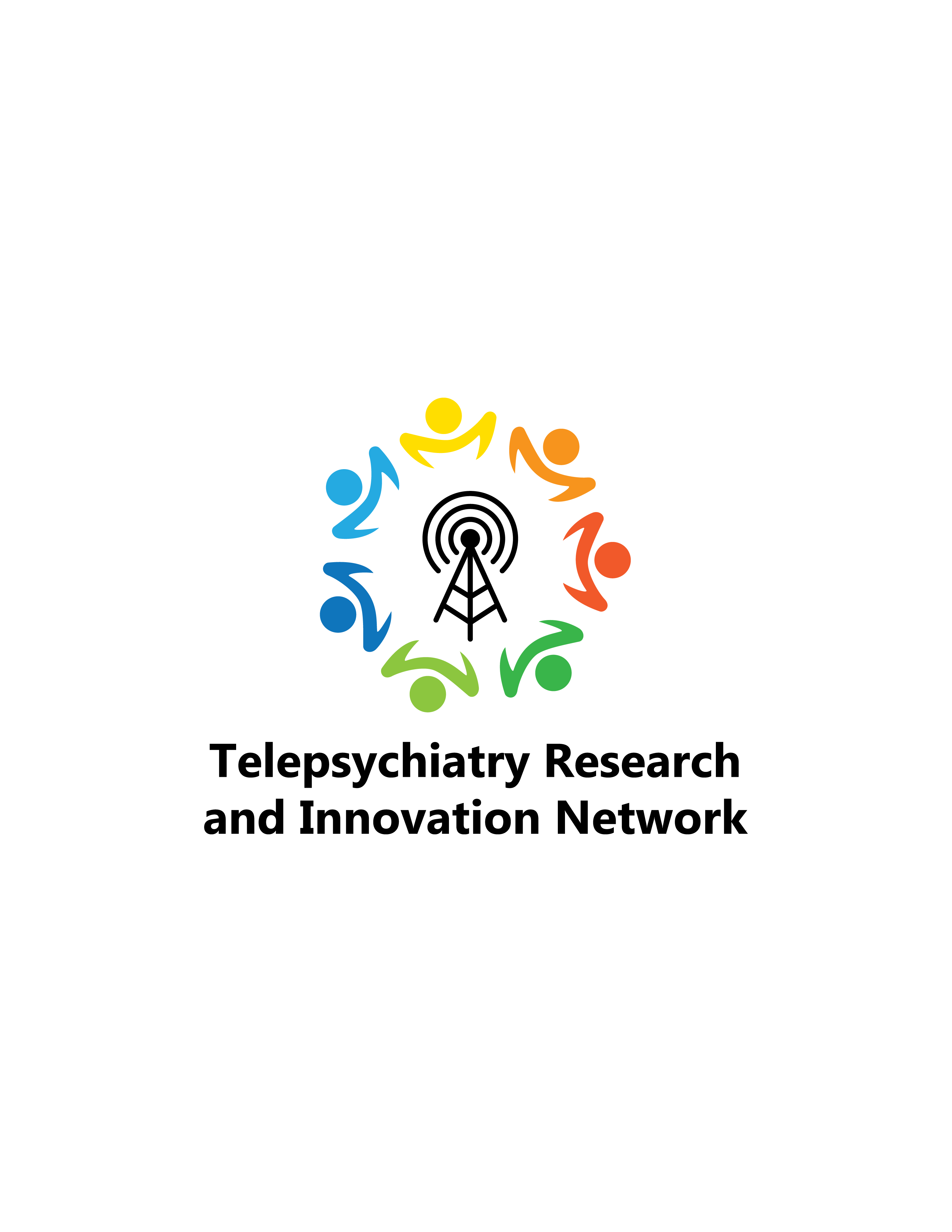

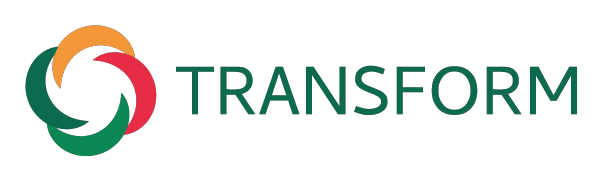


**Transforming Access to Care for Serious Mental Disorder in Slums (TRANSFORM)**

**Work Package 1**

**Topic Guide for Individual Interviews with Caregiver of Person with Severe Mental Disorder**

Thank you for agreeing to talk with us as part of the TRANSFORM study. Your contribution will help us understand more about mental health in this neighbourhood and your role as a Caregiver.

***General Objective:***

*To develop an understanding of the explanatory models of illness and care in relation to SMDs, as well as the barriers and facilitators of collaboration between healers and health professionals from the perspectives of service users, community members, healers and health professionals.*

***Specific Objectives***

*To understand*

1. *Local communities’ perceptions (explanatory models) about SMDs*
2. *the existing understanding about referral pathway for SMDs*
3. *traditional and faith-based healers' treatment strategies (ideas about screening of SMDs)*
4. *perception of the community about collaboration between healers and health professionals to improve the outcomes of persons with SMDs*
5. *Perception of plural healing for SMDs*

Thank you for agreeing to talk with us as part of the TRANSFORM study. Your contribution will help us understand more about the lived experience of mental health in this neighbourhood

**Section 1: General**

1. Please tell us about your family.

Probe:

Relationship to person with SMD, family composition, relationship among family members, number of children, position in the family.

1. Tell us about your living conditions.

Probe:

Describe the place where you live. Who you live with, how long have you lived with them? Who owns the place where you live? How long have you lived here? What is it like to live in this neighbourhood? Difficulties and benefits of living in this neighbourhood?

**Section 2: Perception of Health**

1. What are the types of health problems that people in this neighbourhood experience?

Probe:

Can you give more detail? What do you think are the major causes of illnesses in your neighbourhood? What are the main sources of treatment if someone becomes unwell?

**Section 3: Experiences of mental illness / Local communities’ perceptions (explanatory models) of SMDs**

1. Please tell us about your relative’s mental health condition.

Probe:

What is the name of your relative’s condition? How long has your relative had this condition? Is your relative ill all the time or does the illness come and go?

1. What were the signs that your relative was ill the first time it happened?

Probe:

How did your relative behave? (Probe: acting strangely, seeing or hearing things other people did not, spending time alone, not eating, problems sleeping, crying a lot, sounding and/or looking frightened/sad/angry etc., being very reckless, going naked).

Who noticed the changes first?

1. What do you think causes these illnesses?

Probe:

Spiritual causes (e.g., jinns, evil eye),

Social causes (e.g. unemployment, poverty),

Biological causes (heredity or genetics, brain injury, chemical imbalances),

Victimization (e.g., physical or sexual abuse),

Stress, Loss and grief,

Religious/mystical causes (e.g., divine punishment, fate),

Ingestion of food or substances (e.g. Drugs),

Psychological (e.g., emotional trauma, neglect),

Environmental stressors (e.g., socio-cultural expectations, family issues) anything else?

1. How has your relative’s condition changed over time?

Prompt:

Has the illness got worse or better? How has the symptoms vary over time? Does it come and go?

1. Describe the effects of the illness on your daily life?

What has been the effect on your relative’s life?

Probe:

Impact on livelihoods, family relationships, finances, marriage, caring for children

1. How have people in your neighbourhood responded to your relative’s mental health condition?

Probe:

Offers of support, experiences of stigma/social exclusion.

Has this changed over time?

**Section 4: Pathway to care/ plural healing for SMDs**

1. Please tell us about all the different kinds of treatment you have tried from the first sign of illness to the current day?

Probe:

CHWs/traditional healers/faith healers/clinic/hospital.

When did you seek help? Why did you use these facilities?

How did you decide where to go for treatment?

How does your religious faith influence where you seek help?

How did you get to know about them? [E.g. advised by a family member/friend/religious leader/neighbour, previous experience, reputation of healer).

1. What treatment did your relative receive in each of these places over the course of the illness?

Probe:

Medication, herbal medicines, rituals/exorcism

How effective was the treatment at first and over time?

Why do you think it was/was not effective?

What were the signs that your relative was getting better? (Probe: taking part in daily activities, interacting with other people).

Are you still having any treatment? (Taking medication, observing rituals).

1. What was the cost of using these services?

Probe:

Total amount spent,

Cost of medicines/rituals/services received,

Other types of cost incurred [cash, gifts, donations to the facility or healer, sacrificial animals etc.]

Who paid for this and how? [Probe: Loans, selling assets, savings, family members, etc.]

1. What did you think of the treatment your relative received?

What were the positive and negative aspects?

1. What was your experience of these services?

Probe:

What did you think of the treatment your relative received?

Was he/she ever mistreated at any of these places during the treatment? (E.g. Beatings, chaining, enforced treatment, deprivation of food, etc.).

1. Please tell us how you reported this mistreatment?

Probe:

Did you ever report the mistreatment?

Who did you report to? When did you report? What action did they take?

1. What do you know about the human rights of people with mental illness?

**Section 5: Caregiving**

1. How does your relative contribute to the household?

Probe:

Working for income, helping with chores, childcare etc.

1. What help do you give to your relative in his/her day-to-day living?

Probe:

Livelihood support, help with personal care, keeping him/her safe, giving him/her medicine.

Do you receive payment for the care you offer to the person with mental health condition you care for? [Prompt: If so, how much? Is it in cash or in kind?].

1. Tell us about anyone else helping you with caring for your relative’s mental health condition?

Probe:

Family, friends, government organisation, NGOs, place of worship etc.

1. What other sources of help or support do you think you need?

Probe:

Family members, the government, place of worship, NGO.

Who do you think should provide this?

**Section 6: Impact of the COVID-19 pandemic**

1. How has events during COVID-19 pandemic affected your relative’s mental health condition?

Probe:

Changes in social interaction

Changes observed in his/her symptoms [e.g. mildness or severity of symptoms]

Was he/she able to continue receiving care?

Did it affect the treatment your relative received? [E.g. access to medicines and/or healers, access to support from NGOs/peer support, use of restraints etc.].

1. Tell us any restraint or other human rights abuses your relative experienced because of the pandemic?
2. What concerns do you currently have about the COVID-19 pandemic?

Probe:

Financial, health, anything else?, keeping safe, getting vaccinated, crowded accommodation, shared bathrooms, access to water etc.

1. Is there anything else you would like to tell us?

End of Interview.


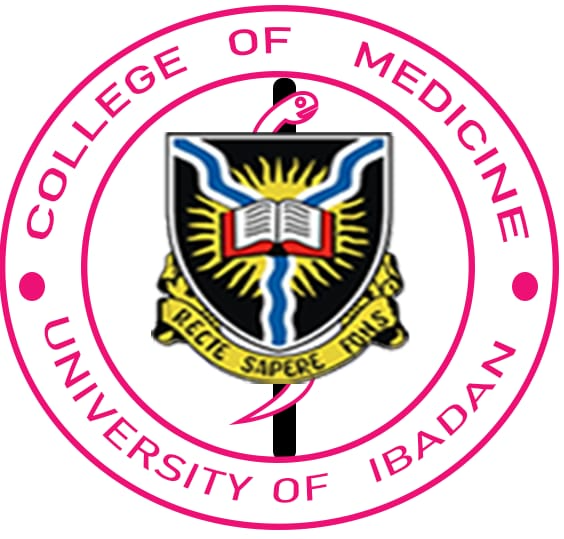

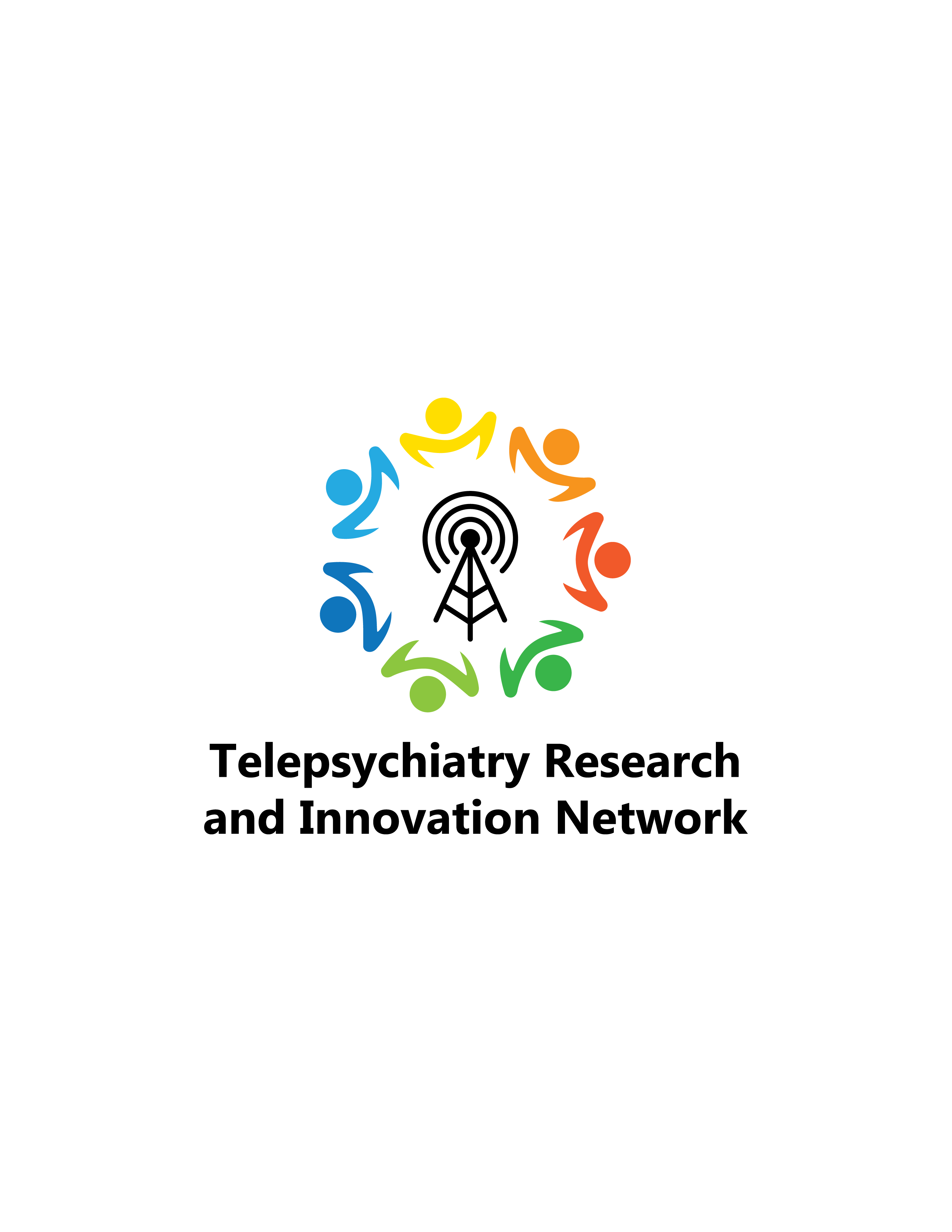

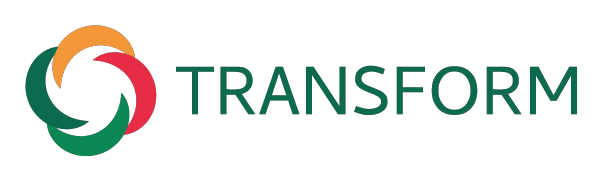


**Transforming Access to Care for Serious Mental Disorder in Slums (TRANSFORM)**

**Work Package 1**

**Topic Guide for Individual Interviews with Persons with Severe Mental Disorders (SMDs)**

Thank you for agreeing to talk with us as part of the TRANSFORM study. Your contribution will help us understand more about mental health in this neighbourhood and your role as a person with SMD.

***General Objective:***

*To develop an understanding of the explanatory models of illness and care in relation to SMDs, as well as the barriers and facilitators of collaboration between healers and health professionals from the perspectives of service users, community members, healers and health professionals.*

Thank you for agreeing to talk with us as part of the TRANSFORM study. Your contribution will help us understand more about the lived experience of mental health in this neighbourhood.

***Specific Objectives***

*To understand*

1. *Local communities’ perceptions (explanatory models) about SMDs*
2. *the existing understanding about referral pathway for SMDs*
3. *traditional and faith-based healers' treatment strategies (ideas about screening of SMDs)*
4. *perception of the community about collaboration between healers and health professionals to improve the outcomes of persons with SMDs*
5. *Perception of plural healing for SMDs*

**Section 1: General Information**

1. Please tell us about your family.

Probes:

Marital status, family composition, relationship among family members, number of children, position in the family

1. Tell us about your living conditions.

Probes:

Describe the place where you live. Who you live with, how long have you lived with them? Who owns the place where you live? How long have you lived here?? What is it like to live in this neighbourhood? Difficulties and benefits of living in this neighbourhood.

1. What are your main sources of income?

Probes:

How often do you get income? Do you do any kind of work to earn money? How often do you work and get paid?

**Section 2: Experiences of mental illness/ Local communities’ perceptions (explanatory models) of SMDs**

1. Please tell us about your mental health condition.

Probes:

How long have you had this condition?

How serious do you think it is?

Is it there all the time or does it come and go?

What is the name of your condition?

1. What were the signs that you were ill the first time it happened?
    Probes:

How did you feel and behave?

Who noticed the changes in your behaviour? [E.g. acting strangely, seeing or hearing things other people did not, spending time alone, not eating, problems sleeping, feeling frightened/sad/anxious/angry etc., crying a lot, feeling very excited/brave etc., feeling restless, being very reckless, going naked].

1. Tell us what you think causes these illnesses?

Probes:

Spiritual causes (e.g., jinns, evil eye),

Social causes (e.g. unemployment, poverty),

Biological causes (heredity or genetics, brain injury, chemical imbalances),

Victimization (e.g., physical or sexual abuse),

Stress, Loss and grief,

Religious/mystical causes (e.g., divine punishment, fate),

Ingestion of food or substances (e.g. Drugs), Psychological (e.g., emotional trauma, neglect), Environmental stressors (e.g., socio-cultural expectations, family issues) anything else?

1. Describe the effects of the illness on your daily life?

Probes:

Impacts on livelihoods, family relationships, finances, marriage, caring for children

1. Describe how people in your neighbourhood have responded to your mental health condition?

Probes:

Offers of support, experiences of stigma/social exclusion, labelling? Has this changed over time?

**Section 4: Pathway to care/** **plural healing for SMDs**

1. Please tell us about all the different kinds of treatment you have tried from the first sign of illness to the current day?

Probes:

Traditional healers/faith healers/clinic/hospital.

When did you seek help? Why did you use these facilities? How did you decide where to go for treatment? How does your religious faith influence where you seek help? How did you get to know about the facilities?

[Probe: advised by a family member/friend/religious leader/neighbour, previous experience, reputation of healer].

1. What treatment did you receive in each of these places over the course of your illness?

Probes:

Medication, herbal medicines, rituals/exorcism

How effective was the treatment at first and over time?

Why do you think it was/was not effective?

What were the signs that you were better? (Probe: taking part in daily activities, interacting with other people)

What kind of treatment are you still having? (Taking medication, observing rituals).

1. What was the cost of using these services?

Probes:

Total amount spent,

Cost of medicines/rituals/services received,

Other types of cost incurred [cash, gifts, donations to the facility or healer, sacrificial animals etc.]

Who paid for this and how? [Probe: Loans, selling assets, savings, family members, etc.]

1. What did you think of the treatment you received?

What were the positive and negative aspects?

1. Describe how you were treated at any of these places?

Probes:

Were you ever treated badly at any time during your treatment [e.g. Beatings, chaining, enforced treatment, deprivation of food, etc.].

1. Please tell us how you reported this mistreatment?

Probes:

Did you ever report the mistreatment? Who did you report to? When did you report? What action did they take?

1. What do you know about the human rights of people with mental illness?

**Section 5: Caregiving**

1. What help does your family give you in day-to-day living?

Probes:

Livelihood support, help with personal care, keeping you safe, giving you medicine, other forms of help received.

1. Tell us other people helping you with your mental health condition.

Prompt:

Family, friends, government organisation, NGOs, place of worship

1. What other help or support do you think you need?

Probes

Who do you think should provide this? [Prompt: family members, the government, place of worship, NGO].

**Section 6: Impact of the COVID-19 pandemic**

**19.** How did events during the COVID-19 outbreak affect your mental health condition?

Probes:

Changes in social interaction

Changes observed in your symptoms [e.g. mildness or severity of symptoms]

Were you able to continue receiving care?

Did it affect the treatment you receive? (E.g. Access to medicines and/or healers, access to support from NGOs/peer support etc.).

20. Tell us any restraint or other human rights abuses you experienced because of the pandemic?

21. What concerns do you currently have about the COVID-19 pandemic?

Probes:

Financial, health, anything else?, keeping safe, getting vaccinated, crowded accommodation, shared bathrooms, access to water etc.

22. Is there anything else you would like to tell us?

End of interview.
